# Public Engagement Strategies in Digital Health Ethics: Protocol for a Scoping Review

**DOI:** 10.2196/86280

**Published:** 2026-03-19

**Authors:** Stella Namuganza, Mariacarla Gadebusch Bondio, Philipp Kellmeyer

**Affiliations:** 1 Institute for Medical Humanities, University Hospital Bonn, Bonn, Germany; 2 Hertie Institute for AI in Brain Health, University of Tübingen, Tübingen, Germany; 3 Data and Web Science Group, School of Business Informatics and Mathematics, University of Mannheim, B6, 26, Mannheim, D-68159, Germany, 49 621181 ext 2422; 4 Human-Technology Interaction Lab, Department of Neurosurgery, Faculty of Medicine, University Medical Center Freiburg, Freiburg im Breisgau, Germany

**Keywords:** digital health, digital health ethics, public engagement, participation, representativeness, scalability, artificial intelligence, AI

## Abstract

**Background:**

The ethical, legal, and social issues accompanying the latest advancements in digital health technologies highlight the need to involve the public in their design, development, and deployment to align with societal values and needs. For public engagement to be meaningful, it should be participatory, inclusive, and scalable. However, studies in participatory digital health do not characterize public engagement strategies in terms of scalability, representativeness, and the extent of participation. Moreover, no reviews have examined how ethical debates shape the design and implementation of public engagement strategies in digital health ethics.

**Objective:**

The objectives of the planned study based on this protocol are to (1) identify approaches used to engage the public in digital health ethics; (2) characterize approaches used in digital health ethics in terms of scalability, representativeness, and extent of participation; and (3) explore the role of ethics in the design and implementation of participatory methods.

**Methods:**

The research will be undertaken using the Joanna Briggs Institute scoping review method. To identify relevant literature, the academic databases PubMed, ScienceDirect, IEEE Xplore, and Web of Science will be searched for articles published from January 1, 2015, to November 30, 2025. All retrieved papers will be uploaded to the Rayyan software. Duplicates will be removed, and subsequently, 2 reviewers will independently screen titles and abstracts, followed by full-text screening using a hybrid verification model. Data will be extracted on a spreadsheet, with rows representing individual studies and columns capturing categories of extracted information.

**Results:**

Currently, the search has yielded 1352 articles; at the title and abstract screening phase, 1291 (95.5%) articles have been excluded and 61 (4.5%) have been included. Anticipated results are the number of studies, bibliographic details of the studies (ie, author, publication year, journal, and country), participant characteristics, digital health technology type, participatory methods, and media, as well as the embodiment of participatory, representative, and scalable characteristics in engagement methods and how ethical debates influence the design of public engagement strategies.

**Conclusions:**

This protocol outlines methodology for a scoping review mapping the characteristics and ethics of public engagement methods to improve participatory and ethically responsible innovation in digital health.

## Introduction

### Background

The convergence of digital health technologies and recent advancements in artificial intelligence (AI) promises substantial improvement in the health care sector. Nonetheless, these innovations are accompanied by ethical, legal, and social concerns [1], which have awakened public skepticism [2] and heightened ethical scrutiny from the public. These dynamics underscore the need to engage the public in the design, development, and deployment of digital health technologies to ensure that innovations align with societal values and needs.

Public engagement is widely recognized as an essential approach for identifying complex human experiences as well as ethical, legal, and social aspects associated with digital health tools [3-7]. Its goals extend beyond resolution of controversy, public education, democratization of science, widening the representation of voices, enabling responsible innovation, and shaping policy [8]. A range of approaches have been used to engage the public in debates on digital health, including consultation of patient organizations [9], educational campaigns, social media [10], and mainstream media outputs (talks, publications, and podcasts), as well as participatory co-design methods [11] drawing on workshops, think‐aloud interviews, focus groups, and surveys to facilitate engagement both in person and online [12].

Two prominent frameworks, ethics by design and embedded ethics, have been proposed to promote the ethical development of digital health tools [13]. They aim to integrate ethical reflection directly into the development and implementation of digital technologies rather than treating ethics as a retrospective or external evaluation. Ethics by design emphasizes the proactive incorporation of ethical values—such as fairness, transparency, and respect for autonomy—into system architectures, design choices, and development processes. Embedded ethics, in contrast, focuses on systematically integrating ethicists or ethical expertise into interdisciplinary research and development teams, enabling continuous ethical reflection throughout the innovation life cycle [14]. Both approaches have been proposed as means to align technological innovation with societal values and are increasingly discussed in the context of digital health and medical AI. These frameworks have been criticized for their top-down orientation, which imposes normative principles externally rather than allowing them to emerge from users’ lived experiences. As an alternative, community-led approaches should actively involve end users or potential end users from the earliest stages of design to ensure that local values and social contexts are embedded during the innovation processes [15]. As different communities acknowledge and push for the agenda of public participation, including research governance [16], there is a shift from simply acknowledging the need for public participation to considering how it can be meaningfully achieved [17]. This entails examining the facets that make public participation meaningful, which include but are not limited to inclusivity, representativeness, empowerment, and scalability [15,18]. When these conditions are met, public engagement in the domain of digital health not only fosters ethical and socially responsible innovation but also contributes to improved health outcomes [19,20].

A preliminary search conducted on July 15, 2025, in PubMed, Scopus, and Web of Science retrieved primary research papers on methods used to engage the public in the context of digital health ethics; however, there are no review papers mapping different methods used to engage the public in digital health ethics and characterizing them in terms of scalability, representativeness, and how participatory they are. Moreover, there are no papers reviewing ethical debates to determine how they influence the design of public engagement strategies. However, the growing practice of public engagement in digital health ethics requires critical inquiry into the quality and standards of the methods used to engage the public.

Therefore, this study will provide a comprehensive overview of the approaches used to engage end users and potential end users in discussions on digital health ethics and characterize the methods in terms of scalability, representativeness, and the extent to which they are participatory. Additionally, the study will determine how ethical debates unfold in the design and implementation of participatory methods. This will establish a foundation for enhancing public participation in digital health ethics. This research aims to foster effective public engagement strategies in the field of digital health ethics. This protocol outlines the methodology and reporting plan for a scoping review aimed at addressing these aims.

### Operational Definition of Terms

In this study, “digital health ethics” refers to the field of practice concerned with addressing ethical, legal, and social issues in the design, governance, development, and implementation of digital health technologies. This includes ethics-by-design approaches, embedded ethics, value-sensitive design, and other structured efforts to operationalize ethical reflection in digital health innovation. It ensures that these tools benefit patients and society while minimizing harm. Key ethical considerations include privacy and data security, algorithmic bias and fairness, informed consent, patient and professional autonomy, transparency and accountability, and equitable access to digital health solutions.

In this study protocol, “articles” refers to records and publications identified through database searching and screening, while “studies” refers to the empirical research reports included in the scoping review and subjected to data extraction and synthesis. Table 1 provides a full list of operational definitions of terms.

**Table 1.** Operational definition of terms.

Term	Definition
Digital health	Encompasses health-related AI^a^, mobile health, telemedicine, electronic health records, online health communities, and big data analytics used to enhance health care and improve health outcomes [21,22].
Public engagement methods	Defined as an organized procedure through which certain members of the public (users, affected communities, citizens, and stakeholders) are invited to contribute or exchange with technology developers their views, knowledge, or decisions about AI or algorithmic systems (eg, workshops and interviews) through media that enable the engagement to happen (eg, face-to-face, social media, and digital platforms) [17].
Participation	Defined as the active involvement of end users in the development and implementation of digital health technologies, including transferring decision-making power to these users [23,24].
Representativeness	The degree to which the participants of an engagement activity, such as a workshop, reflect the broader population they are drawn from in terms of experiences and needs [25].
Scalability	Refers to the expansion of an intervention from a small or controlled setting to a broad real-world application without loss of efficacy [26].

a AI: artificial intelligence.

### Objectives of the Study

The objectives of this study are as follows:

1. To identify approaches (methods and media) used to engage the public in digital health ethics
2. To characterize approaches used in digital health ethics in terms of scalability, representativeness, and extent of participation
3. To explore how ethical debates play a role in the design and implementation of participatory methods

### Research Questions

The following research questions (RQs) correspond directly to the study objectives and guide evidence charting and synthesis:

1. What methods are used to engage the public in digital health ethics?
2. What media or communication channels are used to engage the public in digital health ethics?
3. To what extent are the different public engagement methods truly participatory?
4. How scalable and representative are the methods used to engage the public in digital health ethics?
5. How do ethical debates play a role in the design and implementation of participatory methods?

## Methods

### Overview

The research will be undertaken using the Joanna Briggs Institute (JBI) scoping review method. This framework was first proposed by Arksey and O’Malley [27] and later modified by Levac et al [28] to provide details about what occurs at each stage of the review process. Peters et al [29] further enhanced this approach into the JBI framework to connect eligibility criteria with research objectives. We adopted a scoping review approach because participatory design in digital health is an emerging and conceptually diverse field that benefits from systematic mapping of existing evidence. Therefore, we intend to map the breadth of existing evidence. Given the exploratory nature of our questions and the heterogeneity of the evidence, a scoping review is appropriate to map the literature and systematically identify knowledge gaps. To ensure methodological robustness and transparency, we adopt a systematic approach guided by the *JBI Manual for Evidence Synthesis* for scoping reviews [29]. In addition, the review will be reported in accordance with the PRISMA-ScR (Preferred Reporting Items for Systematic Reviews and Meta-Analyses extension for Scoping Reviews) checklist [30].

### Search Strategy

Title and abstract screening will be conducted using Rayyan (Qatar Computing Research Institute) [31], a web-based software platform designed to facilitate systematic and scoping reviews by enabling independent, blinded screening of records and efficient resolution of reviewer disagreements.

The academic databases PubMed, ScienceDirect, IEEE Xplore, and Web of Science will be searched for studies published from January 1, 2015, to November 30, 2025. The year 2015 marks the onset of three converging developments: (1) the mainstreaming of the Internet of Things and wearables in health care, (2) the first wave of AI-driven digital health applications and policy frameworks (eg, the European Union’s Digital Single Market Strategy), and (3) a growing shift in research funding and governance frameworks toward “public and patient involvement” as a quality criterion [16,32,33]. Together, these mark a distinct phase in the evolution of digital health that justifies our temporal scope.

The search terms in Table 2 will be used to search for papers. Reference lists of relevant studies will also be searched for eligible studies. The search strings were adapted to the specific syntax and indexing conventions of each database (eg, MeSH [Medical Subject Headings] terms in PubMed) rather than applying a single identical string across all platforms.

**Table 2.** Search terms.

Database	Search terms
PubMed, ScienceDirect, and Web of Science	(Digital OR Artificial intelligence) AND participatory ethics Digital participatory codesign AND Health AND Ethics (AI ethics OR ethical AI) AND (co-design OR participatory design) AND (inclusion OR empowerment OR representation) (responsible research and innovation OR RRI) AND (health OR AI in health) AND (participatory OR user involvement) (deliberative democracy OR deliberative engagement) AND (digital health OR health technology) AND (ethics OR value alignment) (AI ethics OR artificial intelligence AND ethics) AND (public engagement OR stakeholder participation) AND (inclusion OR justice OR responsible innovation) (digital health OR e-health) AND (community engagement) AND (ethics OR ethical framework OR value-sensitive design)
IEEE Xplore	Digital participatory codesign AND Health AND Ethics “digital health” OR “health technology” OR “Artificial intelligence AND ethics AND participatory” OR “public engagement” AND health (“responsible research and innovation” OR RRI) AND (health OR “AI in health”) AND (participatory OR “user involvement”)

### Eligibility Criteria

The eligibility criteria were developed using the population, concept, and context framework (Textbox 1). The population comprises end users or potential end users of digital health technologies (patients, health care consumers, or the lay public involved in digital health). The concept is the public engagement strategies in the context of digital health ethics (ethics-by-design, embedded ethics, governance consultations, or other structured efforts to address ethical, legal, and social issues). Context is any setting where digital health technologies are designed or implemented (with no geographic limitations). The search is limited to publications from January 1, 2015, to November 30, 2025.

Textbox 1. **Eligibility criteria.**

**Inclusion criteria**

- Empirical studies using qualitative, quantitative, or mixed methods with primary data collection from end users or potential end users of digital health technologies
- Studies engaging or collecting data from end users (eg, patients, citizens, and consumers) in the context of digital health
- Studies addressing digital health technologies
- Studies incorporating ethical, public engagement, or participatory design elements
- Articles in English

**Exclusion criteria**

- Pure ethics papers, conceptual papers, or reviews (systematic, scoping, or narrative reviews) as we aim to map primary evidence; however, for the background discussion, conceptual or normative papers that propose frameworks or arguments (even if not presenting new data) will be included as these inform research question 5
- Nonscholarly sources lacking primary data, such as newspaper articles, blogs, magazine articles, comments, letters, editorials, and review articles
- Studies that collect data only from non–end users of digital health technologies and do not include end users as participants
- Articles that do not include any explicit discussion of ethical considerations, public engagement activities, or participatory design methods in relation to digital health technologies
- Publications outside the time frame of January 1, 2015, to November 30, 2025, reflecting a period of notable growth in relevant literature
- Research protocols or proposals
- Studies not addressing digital health issues
- Animal studies

Conceptual or normative publications are not included in the scoping review’s data extraction or synthesis and, therefore, are excluded as evidence sources. Such literature is consulted solely to inform the conceptual framing and interpretation of ethical themes identified within the included empirical studies, particularly in relation to RQ 5. Accordingly, the inclusion criteria focus on studies that explicitly intersect public engagement and digital health ethics within the health domain, whereas conceptual contributions on digital ethics or participation alone are used only for contextual framing and are not included in the data extraction phase.

### Selection Process

All retrieved papers from the different databases will be uploaded to Rayyan to ensure a transparent record of all processes and blind screening. Duplicates will be removed, and subsequently, 2 reviewers will screen titles and abstracts. Any disagreements will be resolved through discussions among the 3 reviewers until consensus is reached regarding inclusion or exclusion (interrater disagreements will be documented for transparency). Articles identified as potentially relevant based on title and abstract screening will be retrieved in full text and assessed for eligibility by 1 reviewer, with uncertainties deliberated in team meetings to ensure agreement. Only articles in the English language that the team can reliably understand will be selected. Only studies meeting the inclusion criteria following full-text screening will be retained for the final analysis. A detailed record of the selection process will be reported in the final publication using a PRISMA-ScR flowchart. Reasons for exclusion at the full-text stage will be recorded.

### Data Extraction

A structured data extraction spreadsheet will be developed by 1 reviewer initially in consultation with the review team. The data extraction sheet will be piloted across 2 to 3 studies to ensure that all authors agree on the interpretation of the categories and refine it as needed. Two reviewers will independently chart data from the first few studies and compare results to ensure consistency before 1 reviewer completes charting for the remaining studies (with a second reviewer verifying a sample of the extractions for accuracy). The spreadsheet will be organized with rows representing individual studies and columns capturing categories of extracted information, including bibliographic details, study design, target population, and digital health context (eg, technology type). Additional columns will capture conceptual data aligned with the RQs. Specifically, for RQ 1, data on engagement methods will be collected and charted; for RQ 2, information on engagement media will be extracted; for RQ 3, the degree of participation will be mapped against the ladder of participation (ranging from information, consultation, partnership, and co-design to community-led approaches) [18,34]. For RQ 4, any descriptions of the scale of engagement (eg, number of participants and multisite vs single site) and notes on representativeness (eg, demographics of participants compared to the target population) will be extracted and charted to assess scalability and representativeness. If a study does not explicitly address scalability or representativeness, we will record that these were not mentioned. For RQ 5, conceptual and normative publications will be consulted to interpret how ethical frameworks within digital health ethics practice shape public engagement strategies. Any additional data deemed relevant to addressing the RQs but not captured within these predefined categories will also be recorded and analyzed.

### Analysis and Presentation of Evidence

Data will be analyzed using descriptive and thematic approaches. A descriptive numerical summary of the included studies (eg, by engagement method, country, or technology type) will be presented in tables and figures to provide an overview of the evidence base (Multimedia Appendix 1).

In the qualitative synthesis, findings will be organized by RQ. Engagement methods and media (RQs 1‐2) will be summarized and mapped to their level of participation using a modified model of Arnstein’s ladder of participation [34], which provides a conceptual continuum from information and consultation to partnership and community control. Issues of scalability and representativeness (RQ 4) will be analyzed by comparing the scope and demographic breadth of participation reported across the studies.

For RQ 5, we will conduct a thematic content analysis [35] of ethical debates and frameworks described in the included literature. The analysis will be partly deductive, guided by key constructs from responsible research and innovation [36] and value-sensitive design [37], and partly inductive to capture emergent ethical themes. These may include privacy, fairness, autonomy, trust, and accountability as recurring normative dimensions of public engagement.

Results will be presented as a combination of quantitative summaries (tables and figures) and narrative thematic synthesis. Consistent with scoping review guidance, we will not conduct a formal risk-of-bias assessment as the purpose is to map all relevant evidence irrespective of methodological quality. However, we will note any apparent limitations of the evidence base in the discussion.

The optional consultation stage will take place after data analysis through discussions with digital health ethics experts and community representatives to validate interpretations and enhance practical relevance.

Any protocol amendments made during the review will be documented in the published record, including the date and rationale. Findings will be interpreted within the broader theoretical discourse on participatory and responsible digital health innovation.

### Ethical Considerations

This study analyzes publicly available data and does not involve human participants; therefore, ethics approval was not required.

## Results

**Figure 1.**
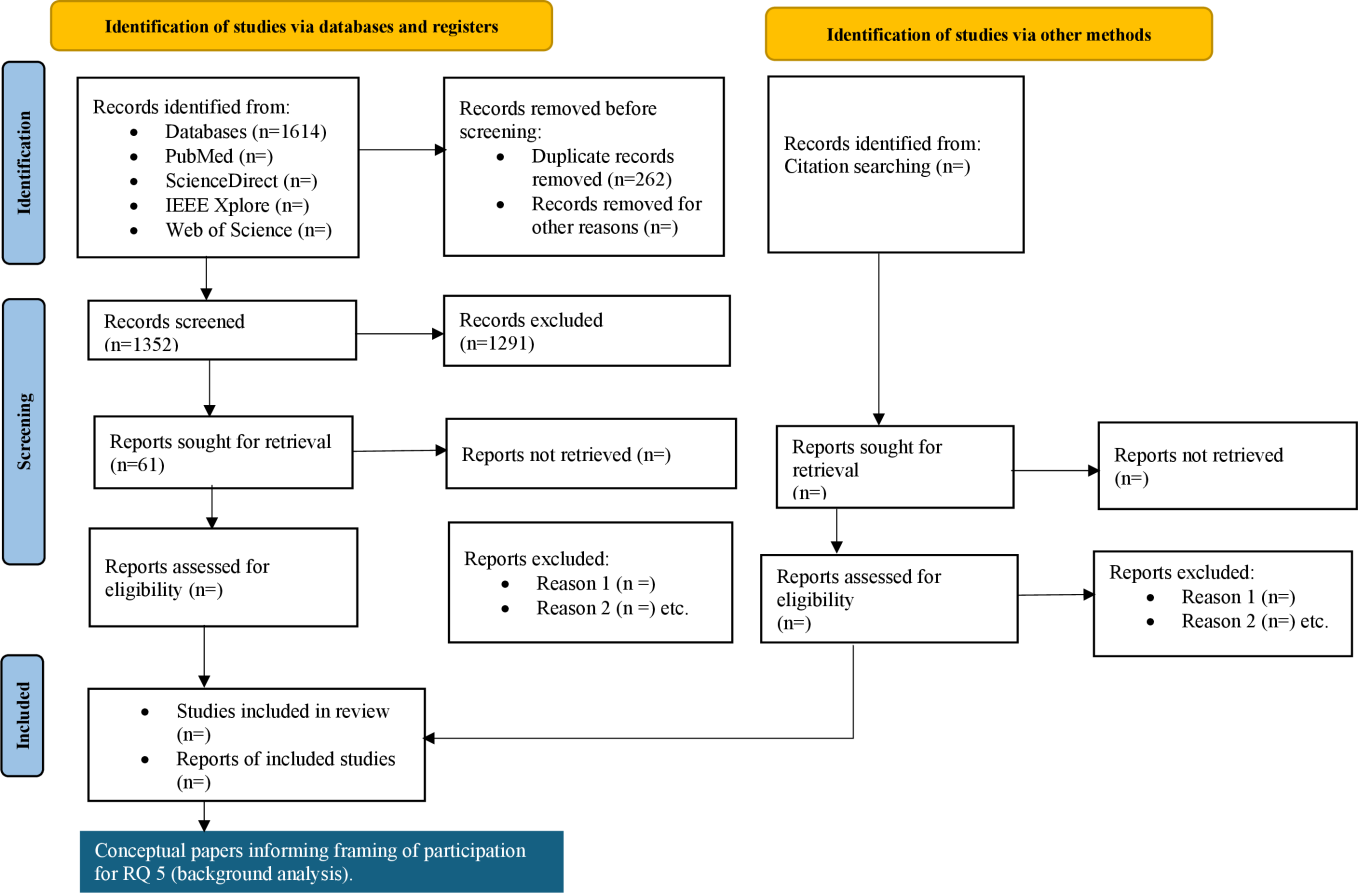
PRISMA-ScR (Preferred Reporting Items for Systematic Reviews and Meta-Analyses extension for Scoping Reviews) flowchart. RQ: research question.

The timeline for this research is as follows: retrieval and screening of articles will be conducted between July 15, 2025, and November 30, 2025; full-text screening will take place between November 15, 2025, and December 15, 2025; and data extraction and analysis will be conducted in January 2026. Figure 1 shows a preliminary version of the PRISMA-ScR flowchart.

Results are expected to be submitted for publication in the first quarter of 2026. Currently, the search has yielded 1352 articles; at the title and abstract screening phase, 1291 (95.5%) articles have been excluded, and 61 (4.5%) have been included. Funding for this scoping review is blinded. Anticipated results are number of studies, bibliographic details of the studies (ie, author, publication year, journal, and country) of the studies, participant characteristics, funding source, digital health technology type, participatory methods, and media. Additionally, we anticipate results on the embodiment of participatory, representativeness, and scalability characteristics by engagement method and how ethical debates influence the design of public engagement strategies.

## Discussion

### Anticipated Findings

This scoping review will provide the first comprehensive mapping of public engagement strategies in digital health ethics. By systematically characterizing engagement approaches according to their participatory depth, scalability, and representativeness, the review will identify methodological gaps and highlight examples of effective, community-led practices while comparing them with previous literature. The findings are expected to inform researchers, developers, and policymakers seeking to integrate participatory and ethically grounded approaches into digital health innovation.

Beyond methodological insights, the review will also contribute conceptually to the discourse on digital health ethics by clarifying how ethical debates, such as those on fairness, transparency, and autonomy, shape the design and implementation of engagement strategies. The resulting synthesis will serve as an evidence base for refining participatory frameworks in digital health research and practice.

### Limitations

Several limitations should be acknowledged. The availability of English-language abstracts and database indexing constrains coverage. Second, the search will rely primarily on published and indexed literature; while targeted gray literature searches will be conducted, relevant reports or policy documents may remain undiscovered. Third, consistent with scoping review methodology, the review will not include a formal critical appraisal of study quality, which may limit the ability to assess the robustness of individual study findings. Finally, because participatory and ethical terminology varies widely across disciplines, some potentially relevant studies may use different descriptors and, thus, be missed despite a comprehensive search strategy.

### Conclusions

This protocol outlines the rationale and methodology for a scoping review that will systematically map public engagement strategies in digital health ethics. By identifying and characterizing participatory approaches across diverse contexts, the review seeks to clarify how ethical considerations are embedded in engagement strategy design and implementation. The anticipated findings will provide an evidence-informed foundation for improving participatory, ethically responsible innovation in digital health.

## Supplementary material

10.2196/86280 Multimedia Appendix 1 Data extraction framework.

## Abbreviations

AI: artificial intelligence
JBI: Joanna Briggs Institute
MeSH: Medical Subject Headings
PRISMA-ScR: Preferred Reporting Items for Systematic Reviews and Meta-Analyses extension for Scoping Reviews
RQ: research question
