# Supplementary material for: Public Engagement Strategies in Digital Health Ethics: Protocol for a Scoping Review
Source: JMIR Res Protoc. 2026 Mar 19;15:e86280. doi: 10.2196/86280 (PMC13002162; doi:10.2196/86280)
Supplement: Multimedia Appendix 1 [file resprot-v15-e86280-s001.docx]

**Table 1:Data extraction Framework**

| **Scoping Review Details** |  |
| --- | --- |
| Scoping Review title: | Public Engagement Strategies in Digital Health Ethics: A Scoping Review |
| Review objectives: | 1. Identify approaches (methods and media) used to engage the public in digital health ethics  2. Characterize approaches used in digital health ethics for scalability, representativeness and extent of participation  3. To explore how ethical debates play a role in the design and implementation of participatory designs |
| Review questions: | 1. What methods are used to engage the public in digital health ethics?  2. What media or communication channels are used to engage the public in digital health ethics?  3. To what extent are the different public engagement methods truly participatory?  4. How scalable and representative are the methods used to engage the public on digital health ethics?  5. How do ethical debates play a role in the design and implementation of participatory designs? |

|  | |
| --- | --- |
| **General Information**   \| Date form completed *(dd/mm/yyyy)* \|  \| \| --- \| --- \| \| Study Title \|  \| \| Name/ID of person extracting data \|  \| \| Reference citation \|  \| \| Notes: \| \| |  |
|  |  |
| **Study eligibility criteria**   \| Study characteristics \| Eligibility criteria / inclusion criteria \| Criteria met (Yes/No) \| \| --- \| --- \| --- \| \| Study design \|  \|  \| \| Population \|  \|  \| \| Concept (participation and digital health ethic) \|  \|  \| \| Context (between January 2015 and December 2025) \|  \|  \| \| Decision \|  \| \| \| Reason for exclusion \|  \| \| |  |
|  | |
| **DO NOT PROCEED IF STUDY EXCLUDED FROM REVIEW**  **Evidence source Details and Characteristics**   \| Characteristics \| Details \| \| --- \| --- \| \| Author \|  \| \| Date of publication \|  \| \| Country \|  \| \| Journal \|  \| \| Target population \|  \| \| Type of digital health technology \|  \| \| Study funding source \|  \| \| Reference to other studies \|  \| |  |
|  |  |
| **Data from Research Questions**   \| RQ1 \| Method/s of engagement \| \| --- \| --- \| \| RQ2 \| Medium of engagement Online/in-person \| \| RQ3 \| Level of being participatory (Score on Arnstein ladder) \| \| RQ4 \| Scalability and representativeness of method (Scale of engagement, sample size, demographic breadth of sample, suggestions on scalability, addressing barriers to scalability) \| \| RQ5 \| Ethical debates and how they influence design of engagement strategies. (data on ethical rationale for engagement for example ethical principles, reference to ethical framework, and ethical approval) \| |  |
|  |  |
|  | |
